# Effect of Sugar versus Mixed Breakfast on Metabolic and Neurofunctional Responses in Healthy Individuals

**DOI:** 10.1155/2017/9634585

**Published:** 2017-06-19

**Authors:** Roberto Codella, Stefano Benedini, Stefano Paini, Andrea Caumo, Michela Adamo, Ileana Terruzzi, Anna Ferrulli, Concetta Macrì, Luca Andreoni, Michele Sterlicchio, Livio Luzi

**Affiliations:** ^1^Department of Biomedical Sciences for Health, Università degli Studi di Milano, Milan, Italy; ^2^Metabolism Research Center, IRCCS Policlinico San Donato, San Donato Milanese, Italy; ^3^Diabetes Research Institute, Metabolism, Nutrigenomics and Cellular Differentiation Unit, San Raffaele Scientific Institute, Milan, Italy; ^4^Neurophysiology Unit, IRCCS Policlinico San Donato, San Donato Milanese, Italy

## Abstract

We investigated the effects of glucose and diverse breakfasts on glucose increment and ghrelin suppression and cognitive processing of sensory information assessed by frontal P300 evoked potentials. In a randomized crossover design, 12 healthy individuals (6M/6F; BMI 22.2 ± 0.4 kg/m^2^; 27 ± 1.3 years, mean ± SEM) underwent 50 g OGTT (A) and 3 breakfasts (B1: milk and cereals; B2: milk, apple, and chocolate cream-filled sponge cake; B3: milk, apple, bread, and hazelnut chocolate cream) to assess plasma glucose-, insulin-, and ghrelin excursions. An electroencephalography was performed before and 100 min after consumption of each load to measure the latency of frontal P300 evoked potentials as index of cognitive performance. Breakfasts B1 and B2 exhibited significantly lower glycemic and insulinemic responses as compared to A. Breakfast B3 exhibited significantly lower glycemic, but not insulinemic response, as compared to A. Final plasma ghrelin inhibition was more pronounced, albeit not significantly, in all breakfasts with respect to A. P300 latency tended to decrease following each of the three breakfasts, but B3 was the only breakfast capable to elicit a statistically significant reduction in P300 latency with respect to A (*p* < 0.01), suggesting ameliorated cognitive performance. Such amelioration was correlated with the 2-hour final inhibition of plasma ghrelin concentration (*r* = 0.61, *p* = 0.01).

## 1. Introduction

Over recent decades, the increasing phenomena of overnutrition and obesity can be attributed to the inadequate levels of physical activity and poor dietary regimens [1]. Lifestyle improvements, including regular physical activity, high-quality diets, and sufficient sleep, are commonly advocated to control weight and prevent the development of obesity [2]. Despite efforts, knowledge on the pathophysiology of obesity is still incomplete and a control on obesity incidence is far to be reached [2, 3]. In fact, several factors are involved in this condition: social behavior and environment, genetic, metabolic, and psychological features [4–7]. Among the healthy habits, mounting research compellingly shows the importance of eating breakfast on a regular basis [6, 8–10]. Breakfast plays a major role in energy homeostasis with its contribution in both caloric and nutrient intake [11, 12]. Surprisingly, findings from nationally representative surveys indicate that breakfast consumption is on the decline [13–16].

Breakfast consumption has been associated with a better global nutritional quality [17, 18], reducing the risk of developing obesity in both adult and pediatric populations [19]. On the contrary, breakfast omission has been recognized as a major risk factor for obesity and prospective weight gain [20, 21]. Observational studies carried out in Europe have shown that children and adolescents who eat breakfast have a reduced risk of becoming overweight or obese and have a lower BMI compared with those who skip breakfast [22]. This means that breakfast is of relevance in a healthy regimen, ensuring a virtuous control of body weight and appetite sensations throughout the day. In addition, individuals who eat breakfast have more chance to meet international dietary recommendations [23, 24], integrating their diet with a proper contribution of vitamins, minerals, and fibers [25–28]. Furthermore, breakfast caloric content ought to be matched with morning/daily energy expenditure in order to accomplish attention challenges and physical tasks [29–31]. Beyond the caloric load, the effects of breakfasts may vary because of macronutrient composition [32], glycemic index [33, 34], and hormonal responses [35]. For instance, ghrelin regulation of satiety is a potential target for treating obesity given its recognized involvement in energy homeostasis [36–38].

Besides the above-mentioned nutritional and metabolic benefits, breakfast consumption has also been related with better mood [39] and enhanced cognitive performance [40]. It has been shown in middle and older aged adults that breakfast consumption improves mood and late-morning cognitive functioning [32]. Conversely, breakfast omission can negatively affect children and adolescents' cognitive ability [41]. While these studies have been concerned with the chronic effects of breakfast consumption, relatively little is known about its acute effects on cognitive functioning. In addition, since previous studies focused on either the metabolic or the cognitive effects of breakfast consumption mostly separately, the possible interactions between such effects have received little attention [42, 43].

We concerned ourselves with the nature of the interactions between the acute metabolic and cognitive effects of breakfast consumption and set out a study to provide some insights into this connection. With this purpose in mind, we selected the most representative combination of foodstuffs from the large-scale Italian market to investigate the effects of glucose alone, or each of three breakfasts, on both the metabolic milieu and the cognitive functioning of healthy subjects. Specifically, glucose, insulin, and ghrelin time courses were monitored, while cognitive processing of sensory information was assessed by frontal P300 evoked potentials.

Cognitive evoked potentials, particularly the P300 component, provide a means of measuring the cognitive processing of sensory information [44]. These potentials are usually detectable when subjects move their attention to a stimulus identified as relevant. This generates a P300 wave. P300 is a wide positive depression, which has latency between 250–350 ms, and it is mainly expressed in frontal (FZ), central (CZ), and parietal scalp (PZ) regions. P300 latency is used to classify stimulus speed: it augments as cognitive performances worsen and vice versa. P300 may be evoked by different stimuli: somesthetic, visual, or acoustic. It has been established that P300 event-related brain potential (ERP) may be sensitive to metabolic state and appetite, and it may interfere with cognitive performance [45, 46]; however, the intimate mechanisms behind these actions remain to be elucidated.

The aim of this study was to investigate how three different breakfasts containing either cereals and milk (B1), or milk, apple, and chocolate cream-filled sponge cake (B2), or milk, apple, bread, and hazelnut chocolate cream (B3), as compared to glucose, affected the metabolic response, the sense of satiety, and the cognitive performance in healthy subjects. To this end, healthy young volunteers underwent an oral glucose tolerance test (OGTT) and three breakfast test-meal loads. Glycemia, insulinemia, and ghrelinemia were measured following OGTT and each breakfast consumption. In an extra experiment, an electroencephalography (EEG) was performed before and after the ingestion of each load. During the EEG, acoustic stimuli were used to generate brain evoked potentials. The latency of the P300 frontal (FZ) evoked potentials was used as marker of cognitive performance.

To our best knowledge, this is one of the few studies combining the metabolic with the neuroelectric assessment of different breakfast intakes.

## 2. Materials and Methods

### 2.1. Study Subjects and Inclusion Criteria

Twelve healthy subjects (6M/6F; 22.2 ± 0.4 BMI kg/m^2^; 27 ± 1.3 years old) on a stable diet, with normal glucose tolerance and no dyslipidemia (according to ADA and ATPIII-NCEP guidelines) were enrolled for this study (Table 1). The study was approved by the Ethical Committee of the “Università degli Studi di Milano.” All subjects signed a written informed consent prior to participation, according to the Declaration of Helsinki. All the procedures used complied with the Good Clinical Practice (GCP) principles.

### 2.2. Research Design

This study was carried out in a randomized-crossover fashion at the Endocrinology and Metabolic Diseases Division, IRCCS Policlinico San Donato (San Donato Milanese, Italy). Each subject underwent two experimental protocols in order to assess metabolic and electrophysiological responses, respectively. The metabolic protocol consisted of one oral glucose tolerance test (50 g OGTT: A) and 3 breakfast meal tolerance tests [one for each breakfast tested: B1 = milk (125 ml) and cereals (30 g); B2 = milk (220 ml), apple (200 g), and chocolate cream-filled sponge cake (30 g); B3 = milk (125 ml), apple (150 g), bread (50 g), and hazelnut chocolate cream (15 g)]. Breakfasts were selected among the most representative ones from the large-scale Italian foodstuff market. In detail, we evaluated three breakfast models exemplified by *Italian recommended daily dietary intake* LARN (featured by the *Italian Society of Human Nutrition*, SINU, and the *Italian National Institute for Research on Food and Nutrition*, INRAN) according to a daily dietary intake of 2000 kcal. These models of breakfast envisaged nutritional composition of three types of breakfasts commonly consumed in Mediterranean countries. For each model, the specific nutrient content was calculated and compared with optimal dietary intake and ratios provided by LARN [47]. Italian guidelines on healthy nutrition recommend to introduce 15–20% of the total daily calorie intake, during the first meal of the day. Thus, the three breakfast models ranged from 8.5% (B1) to 16% (B2) and 18% (B3) of an optimal daily intake.

In the neurofunctional protocol, prior and upon consumption of each load (A, B1, B2, and B3), an EEG was performed. Subjects were admitted to hospital at 8:30–09:00 h after overnight fasting (~12 h), and they were not allowed to eat anything during either tolerance tests or EEG. This routine was repeated in 8 experimental days (4 for each protocol).

### 2.3. Breakfast-Meal Tolerance Tests

Breakfasts (B1, B2, B3, Table 2), or 50 g glucose load (A) were consumed in a randomized order on separate days ~2 weeks apart, to ascertain lipidemia, plasma glucose, and plasma hormone profile. Either breakfasts or glucose were administered at ~09:00 h and eaten completely within 10 min, in front of the physician. Blood samples were collected at −15 and 0 min; thereafter, the subjects received either a breakfast-test meal or 50 g glucose. Hence, additional blood samples were obtained at 15, 30, 45, 60, 75, 90, and 120 min after the ingestion. Glucose was dissolved in an aqueous solution whereas breakfasts were eaten within 10 min.

### 2.4. Analytical Methods

The total amount of blood drawn for each glucose/breakfast tolerance test was about 58.5 mL for each participant. All blood samples were placed on ice until plasma or serum was separated by centrifugation at 4°C (within 1.5 h from sampling). All plasma and serum aliquots were frozen at −60°C for later analysis. All the samples were measured in duplicate. Aliquots of blood to measure ghrelin were collected in test tubes containing EDTA. Plasma ghrelin level (total) was measured through the enzyme-linked immunosorbent assay (ELISA, Merck Millipore, Darmstadt, Germany). Free-insulin was dosed by a highly specific 2-site monoclonal antibody-based immunosorbent assay (ELISA; Dako Diagnostics, Cambridgeshire, UK). The lipid profile and NEFA were measured through the immunoenzymatic technique.

### 2.5. Cognitive Test by EEG

Before and 180 min after glucose/breakfast consumption, an EEG was performed to record P300 wave evoked potentials. Five electrodes connected to the neural machine (Synergy, Lubiana, Slovenia) were placed on scalp A1, A2, CZ, PZ, and FZ regions, as described elsewhere [48, 49]. In this test, a “frequent” acoustic stimulus has been presented to the patient repetitively. A 20% proportion of these stimuli was randomly replaced by a “rare” one, and the averaged response to these “rare” stimuli was recorded as the actual P300 potential. The name “P300” refers to the major positive peak of the response. The P300 latency has normal values ranging 250–350 ms in young, healthy subjects. Latency is inversely related to cognitive functions: it decreases with increasing cognitive performance and vice versa. To our purposes, the latency of the P300 FZ potentials was recorded and subsequently analyzed. During the experiment, subjects were told to silently count the number of “rare stimuli” and they were asked to refer that total number at the end of the testing.

### 2.6. Statistical Analysis

The areas under the curves (AUCs) of plasma glucose and insulin concentration, the incremental glucose peak, and the final inhibition of ghrelin concentration (i.e., the averaged 90- and 120 min ghrelin concentrations minus the fasting ghrelin level) were used as summary measures of the metabolic responses resulting from the intake of either glucose or one of the 3 breakfasts. The P300 latency incremental values (i.e., the differences between the P300 latency measured 180 min after glucose/breakfast consumption and the P300 latency measured before consumption) were used to assess the effect of glucose/breakfast intake on cognitive performance.

The comparison among studies was performed using repeated measures ANOVA with Greenhouse-Geisser correction followed by the Tukey-Kramer post hoc test (every mean was compared with every other mean). Pearson's correlation test and linear regression analysis were also conducted between the inhibition in plasma ghrelin and the decrease in P300 latency. All data were represented as mean ± SEM. A *p* value less than 0.05 was considered statistically significant. Analyses were carried out with the Statistical Package SPSS version 22 for Mac (Armonk, NY, USA; IBM Corp.), Excel 2011 (Redmond, WA, USA; Microsoft), and GraphPad Prism 7 (San Diego, CA, USA).

## 3. Results

### 3.1. Metabolic Data

#### 3.1.1. Glycemia

**Figure 1:**
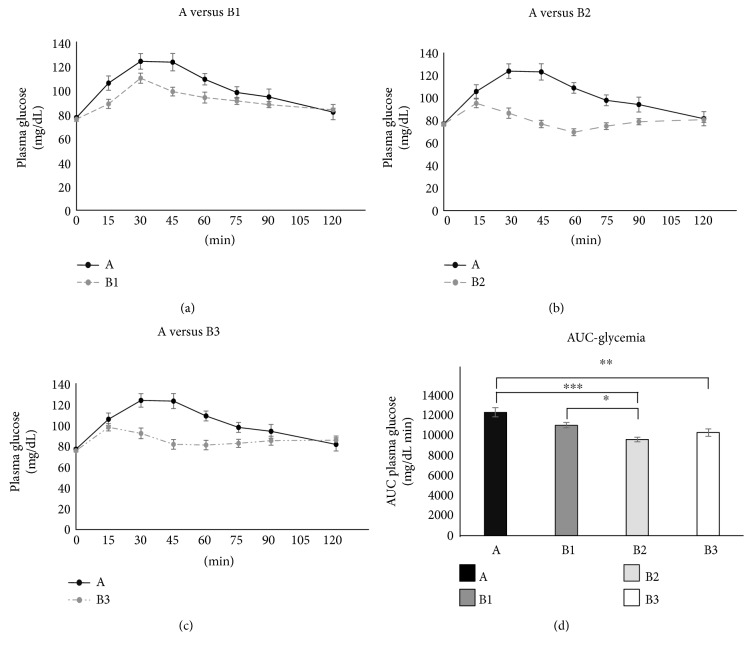
The time courses of glucose concentrations following breakfasts (a) B1, (b) B2, and (c) B3, respectively. For the sake of comparison, each of the three panels also displays the time course of glucose concentration measured after glucose ingestion (black line, glucose). (d) The AUC of glycemia for each test. ^∗^*p* < 0.05; ^∗∗^*p* < 0.01; ^∗∗∗^*p* < 0.001.

Fasting plasma glucose was within the normal range in all subjects (77 ± 1.3 mg/dL prior to test A). As for the comparison among the incremental peak values of glycemia (Figure 1(a), 1(b), and 1(c)), the repeated measures ANOVA with Greenhouse-Geisser correction resulted significant (*p* < 0.001) and the Tukey-Kramer post hoc test revealed that B1 (36.2 ± 3.8 mg/dL), B2 (19.6 ± 2.9 mg/dL), and B3 (27.3 ± 4.0 mg/dL) had significantly smaller glucose incremental peaks than A (55.9 ± 6.1 mg/dL) with *p* values associated to such post hoc comparisons that were *p* = 0.04, *p* < 0.001, and *p* = 0.002, respectively.

As for the comparison among the glycemic AUC values (Figure 1(d)), the repeated measures ANOVA with Greenhouse-Geisser correction was significant (*p* < 0.001) and the Tukey-Kramer post hoc test showed that B2 had smaller glucose AUC than A (*p* < 0.001) and B1 (*p* = 0.048), while B3 had smaller glucose AUC than A (*p* = 0.003).

#### 3.1.2. Insulinemia

**Figure 2:**
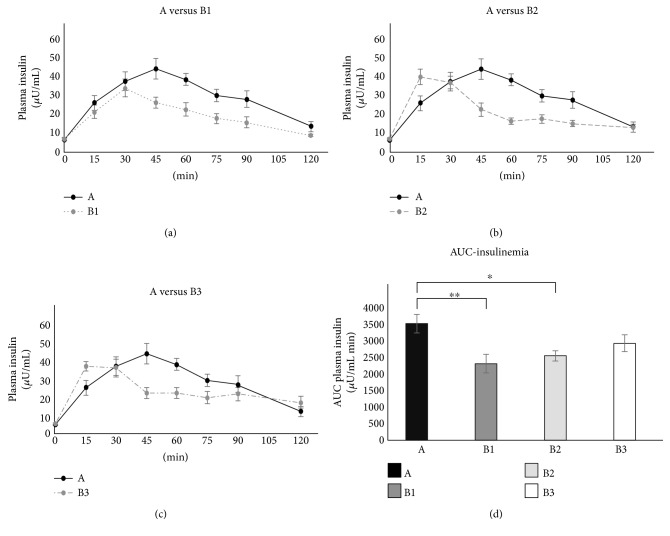
The time courses of insulin concentrations following breakfasts (a) B1, (b) B2, and (c) B3, respectively. For the sake of comparison, each of the three panels also displays the time course of insulin concentration measured after glucose ingestion (black line, glucose). (d) The AUC of glycemia for each test. ^∗^*p* < 0.05; ^∗∗^*p* < 0.01.

Fasting plasma insulin was within the normal range in all subjects (5.25 ± 1.49 *μ*U/mL prior to test A). All three commercial breakfasts showed somewhat smaller insulin excursions with respect to the one elicited by glucose administration (Figure 2(a), 2(b), and 2(c)). As for the comparison among the insulinemic AUC values (Figure 2(d)), the repeated measures ANOVA was significant (*p* < 0.001) (without requiring any correction for the sphericity condition) and the Tukey-Kramer post hoc test showed that both B1 and B2 had smaller insulin AUCs than A (*p* = 0.002 and *p* = 0.016, resp.).

#### 3.1.3. Ghrelinemia

**Figure 3:**
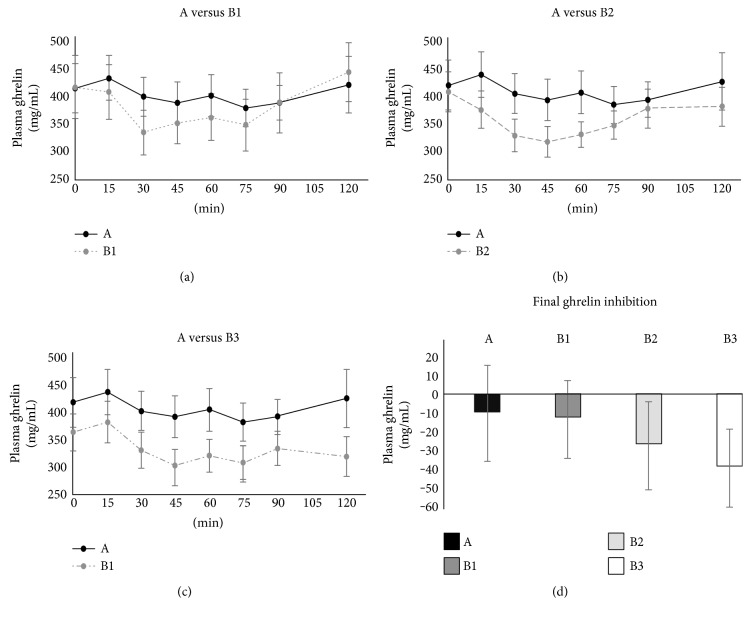
The time courses of ghrelin concentrations following breakfasts (a) B1, (b) B2, and (c) B3, respectively. For the sake of comparison, each of the three panels also displays the time course of ghrelin concentration measured after glucose ingestion (black line, glucose). (d) The final (90–120 min) ghrelin level (expressed as change with respect to the baseline) for each test. None of the differences among the three breakfasts (B1, B2, and B3) and glucose ingestion (A) achieved the statistical significance.

Fasting plasma ghrelin was within the normal range in all subjects (415.5 ± 5.2 pg/mL prior to test A). After glucose/breakfast ingestion, the 2-hour plasma ghrelin attained the lowest level in B3 (332.1 ± 30.2 pg/mL), but the decrement with respect to A (420.6 ± 44.2 pg/mL) did not achieve the statistical significance (Figure 3(c)).

### 3.2. Electrophysiological Data

#### 3.2.1. P300 Event-Related Potentials (ERP)

**Figure 4:**
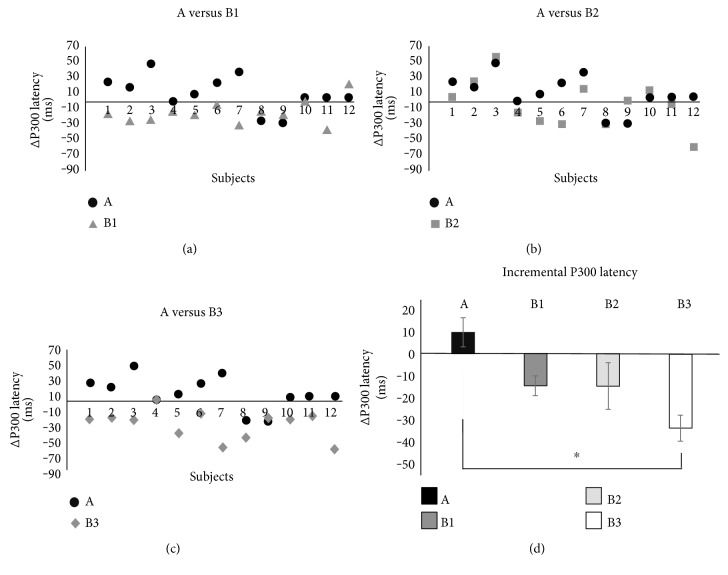
The P300 latency incremental values measured in each of the twelve subjects in association to breakfasts (a) B1, (b) B2, and (c) B3, respectively. For the sake of comparison, each of the three panels also displays P300 latency incremental values measured in association to glucose ingestion (black dots, glucose). (d) The means of the P300 latency incremental values for each test. Of note is that a negative value stands for a peak anticipation. ^∗^*p* < 0.01.

The electrophysiological results are displayed in Figure 4. We reported the P300 latency incremental values measured in each individual subject for each of the three breakfasts (Figure 4(a), 4(b), and 4(c)). For the sake of comparison, each of the three panels also displays the individual P300 latency incremental values measured in association with glucose intake. The means of the P300 latency incremental values are shown in Figure 4(d). All of the tested breakfasts showed some degree of reduction in the P300 latency with respect to glucose. The repeated measures ANOVA with Greenhouse-Geisser correction was significant (*p* < 0.01) and the Tukey-Kramer post hoc test showed that the only significant difference among means was the one between breakfast B3 and glucose (−33.6 ± 5.9 ms versus 9.7 ± 6.6 ms, *p* < 0.01).

### 3.3. Correlation and Linear Regression

**Figure 5:**
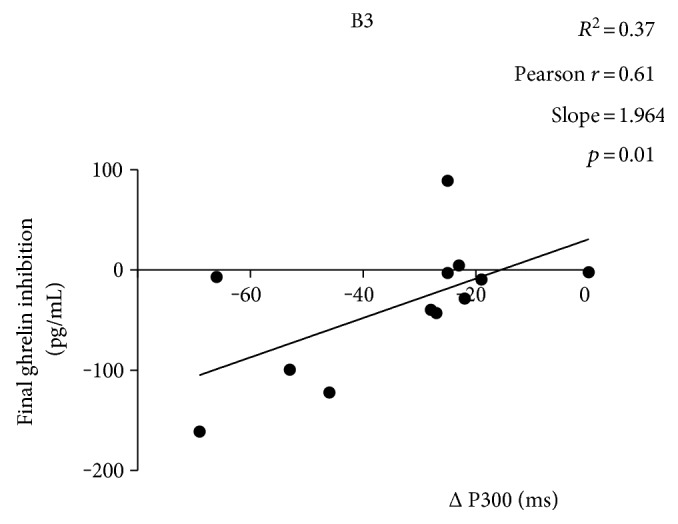
Results of the linear regression analysis between plasma ghrelin inhibition and P300 latency incremental values. The two variables were positively correlated only after consumption of B3 (*r* = 0.61 with *p* = 0.01; slope coefficient = 1.96 pg/mL per ms).

Final (i.e., 90–120 min) plasma ghrelin inhibition and P300 latency incremental values were positively correlated after consumption of B3 (*p* = 0.01; *R*^2^ = 0.37; slope coefficient = 1.96 pg/mL per ms) (Figure 5). No significant correlations/regression slopes were found when the same analysis was repeated for glucose and the other two breakfasts.

## 4. Discussion

In this study, we compared the assumption of three differently composed breakfast meals to a 50 g glucose load to evaluate plasma glucose, insulin, and ghrelin responses in healthy individuals. Along with these metabolic effects, to understand whether other factors may underpin breakfast as a healthy eating behavior, we explored the related neurofunctional effects by measuring frontal P300 wave evoked brain potentials. With these three models of breakfast, we aimed at considering the major contributors of a healthy diet: bread and cereals, as the major source of complex carbohydrates; milk, as a major source of protein, lipid, and calcium; apple as source of fiber, minerals, and vitamin; and chocolate for its hedonistic palatability although in a portion of limited amount of lipids.

We found that each of these breakfasts was able to elicit lower glycemic and insulinemic responses, even with greater carbohydrate content, as compared to the glucose load. B1, B2, and B3 breakfasts displayed a tendency to maintain the sense of satiety via ghrelin inhibition. Furthermore, hazelnut chocolate cream breakfast (B3) consumption improved cognitive performance, assessed by processing of sensory information through EEG. It is of interest to notice that, when the nutritional facts (i.e., energy load and carbohydrate content, Table 2), the metabolic results (i.e., ghrelin inhibition, Figure 3) and the neuroelectric results (i.e., P300 latency, Figure 4) are examined together, a recognizable pattern emerges. All these factors changed almost stepwise from A to B3, thus leading to the hypothesis that the progressive increase in the energy load and carboydrate content from A to B3 produced almost proportional metabolic and electrophysiological effects. This, however, remains a matter of speculation, and further studies will be needed to elucidate this issue.

Although there is a plethora of data examining diversified breakfast behaviors and their acute effects on postprandial metabolic responses, studies analyzing altogether hormonal and neurophysiological features have received considerably less attention. The first meal of the day should represent a healthy food habit for adults and also for children, who crucially need the ability to tightly focus their attention during the early morning school hours. Not only regular breakfast omission is associated with obesity [20, 21], CVD [50], and type 2 diabetes [51, 52] but an appropriate breakfast should contain a blend of macronutrients minimizing insulin excursions and ghrelin levels to foster a hormone milieu not predisposing to obesity.

We previously demonstrated that some isoglucidic snack foods attain a favorable glycemic profile combined with a neatly suppressed plasma ghrelin concentration in the 3-hour postabsorptive status [35]. In analogy with that hazelnut chocolate cream-filled snack, we presently found that B3, a hazelnut chocolate cream-based breakfast, had a subtle excursion of glycemia, with a smaller glucose AUC than the control, and tended to better inhibit final plasma ghrelin 2 hours after breakfast ingestion. Ghrelin decline is not only a biomarker of satiety thus representing an important signal in energy homeostasis, but it may mediate reward responses to food [53], with high significance for the complex and multifaceted pathophysiology of obesity. Moreover, according to the P300 ERP results, B3 breakfast was accompanied by a significant anticipation of the processing information, which equals to a putatively improved cognitive performance with respect to control. More specifically, a significant association was ascertained between final ghrelin inhibition and P300 incremental values, confirming that, after ingestion of B3, subjects were able to better suppress ghrelin and anticipate sensory signals (FZ potentials). Although this correlation cannot be interpreted tout court as the result of a casual relationship between ghrelin suppression and enhanced cognitive performance, nevertheless, this scenario points to the notion that B3 assumption elicited more desirable responses, from both metabolic and neurofunctional standpoints.

Breakfasts have neurofunctional relevance for the daily challenges. The adequate nutritional status is a prerequisite for ensuring efficient learning ability and performance of organized behavior. Several studies document that poor nutritional status can indeed disturb brain function and, even in conditions of appropriate nutritional status, the brain can be very sensitive to acute fluctuations in glucose availability [41]. P300 ERP component is a robust neurophysiological marker of cognitive control-related processing [54, 55]. It has formerly been reported that the frontal (FZ) aspect of the novelty P300 reflects processes related to orienting [49]. In other words, when stimuli are “novel”, in addition to more posterior generators, the anterior aspect of this system is activated. It is as bringing an event to consciousness for subsequent evaluation and appropriate action. This is of paramount interest if we consider adolescent students' learning outcomes.

Among the tested breakfasts, the cereal-based B1 induced the highest glucose response, albeit not significantly. However, all three breakfasts were characterized by smaller glycemic AUCs compared to the control one, suggesting that breakfast composition was not deleterious in respect to the glycemic responses, even when the total amount of carbohydrates was superior than 50 g glucose control. Orchestrating an optimal energy- and macronutrient-model of breakfast is a pivotal issue for food industries committed in sorting out a healthy dietary regimen, fulfilling requirements and recommendations of the major international health agencies and policy makers. Certainly, it would be a limited prospective to rely on the mere glycemic index as a sole guide towards that purpose. Furthermore, during breakfasts, families may be gathering and have the opportunity to promote and transmit healthy habits of sociality and salutary nutrition [56].

Insulinemic responses elicited by the three breakfasts were contained, as indicated by the smaller AUCs with respect to control. This is desirable, considering that postprandial hyperinsulinemia could trigger hunger and promote adiposity and weight gain.

In an integrative view, synergistic feeding-related effects may obviously exist, influencing the interplay between metabolic and neurofunctional mediators. Nevertheless, to a certain degree, an advisable correction of nutritional deficiencies or imbalance could lead to measurable improvements. Varying the choice from the three models of breakfasts may be ideal in a balanced dietary daily regimen.

## 5. Conclusions

In summary, in the present study, we investigated the interactions between the acute metabolic and cognitive effects elicited by three different breakfast types. The main finding was that the breakfast consisting of milk, apple, bread, and hazelnut chocolate cream was the only one capable to produce a significant enhancement of the cognitive performance, which resulted linearly related with the degree of ghrelin inhibition. Although it remains to be elucidated which factor, whether the breakfast caloric content or one of its ingredients, plays the key role in this favorable result, we believe that our findings may contribute to improve breakfast habits in relation with morning attention requirements.

## Figures and Tables

**Table 1:** Anthropometrical and clinical characteristics of the study subjects.

	Mean ± SEM
Sex (M/F)	6M/6F
Age (y)	27 ± 1.3
Weight (kg)	67.6 ± 3.7
Height (m)	1.74 ± 0.03
BMI (kg/m^2^)	22.2 ± 0.4
Fasting glucose (mg/dL)	77 ± 1.25
2-hour glucose (mg/dL)	94 ± 6.61

**Table 2:** Breakfast nutritional facts.

Nutritional facts	Glucose	Breakfasts		
	A	B1	B2	B3
Energy (kcal)	190	170.5	329.2	350.5
(kJ)	795.5	724.8	1389.2	1481.2
Proteins (g)	0	6.2	11	9
Carbohydrates (g)	50	31.5	51.8	61.4
Sugars (g)	50	8.5	44.9	35.1
Fat (g)	0	2.2	9.5	8.3
Saturated fats (g)	0	1.4	4.7	3
Monounsaturated (g)	0	0.35	0.57	0.524
Polyunsaturated (g)	0	0.13	0.175	0.604
Fiber (g)	0	1.14	5.58	8.7
Vitamin A (*μ*g)	0	148.25	28.6	16.25
Vitamin B3 niacin (mg)	0	7.39	0.31	2.44
Vitamin C (mg)	0	7.85	11.4	8.4
Vitamin B2 riboflavin (mg)	0	0.984	0.390	0.372
Sodium (mg)	0	348.15	267.2	402.9
Calcium (mg)	0	146.2	303.2	496
Potassium (mg)	0	212.45	546.2	412.7
Iron (mg)	0	8.76	0.35	2.69

## Abbreviations

AUC: Area under the curve
BMI: Body mass index
CVD: Cardiovascular disease
EEG: Electroencephalography
ERP: Event-related potential
HDL: High-density lipoproteins
LDL: Low-density lipoproteins
NEFA: Nonesterified fatty acids
OGTT: Oral glucose tolerance test.
